# Phase III Preclinical Trials in Translational Stroke Research: Community Response on Framework and Guidelines

**DOI:** 10.1007/s12975-016-0474-6

**Published:** 2016-06-14

**Authors:** Johannes Boltze, Daniel-Christoph Wagner, Nils Henninger, Nikolaus Plesnila, Cenk Ayata

**Affiliations:** Fraunhofer Research Institution for Marine Biotechnology and Institute for Medical and Marine Biotechnology, University of Lübeck, Mönkhofer Weg 239a, 23562 Lübeck, Germany; Fraunhofer Institutes for Cell Therapy and Immunology, Leipzig, Germany; Department of Neurology, University of Massachusetts Medical School, Worcester, MA USA; Institute for Stroke and Dementia Research, University of Munich Medical Center, Munich, Germany; Neurovascular Research Lab, Massachusetts General Hospital and Harvard Medical School, Boston, MA USA

**Keywords:** Animal models, Experimental, Focal ischemia, Randomized controlled trials, Multicenter preclinical trials, Regeneration and recovery

## Abstract

The multicenter phase III preclinical trial concept is currently discussed to enhance the predictive value of preclinical stroke research. After public announcement, we collected a community feedback on the concept with emphasis on potential design features and guidelines by an anonymous survey. Response analysis was conducted after plausibility checks by applying qualitative and quantitative measures. Most respondents supported the concept, including the implementation of a centralized steering committee. Based on received feedback, we suggest careful, stepwise implementation and to leave selected competencies and endpoint analysis at the discretion of participating centers. Strict application of quality assurance methods is accepted, but should be harmonized. However, received responses also indicate that the application of particular quality assurance models may require more attention throughout the community. Interestingly, clear and pragmatic preferences were given regarding publication and financing, suggesting the establishing of writing committees similar to large-scale clinical trials and global funding resources for financial support. The broad acceptance among research community encourages phase III preclinical trial implementation.

**Electronic supplementary material:**

The online version of this article (doi:10.1007/s12975-016-0474-6) contains supplementary material, which is available to authorized users.

*“There are many aspects to P3PT that are important including quality standards, assessment criteria, funding, data management, and choice of participating centres. All of these will have to be rigorously thought through, and dealt with before P3PT goes ahead.”* – selected statement from an individual commenting on the P3PT concept

## Introduction

The concept of multicenter ‘phase III’ preclinical trials (P3PT) for the evaluation of neuroprotective strategies is suggested to overcome the translational roadblock in stroke research [1]. Importantly, it is not meant to replace exploratory scientific work as usually performed by individual laboratories (‘phases I and II’), but represents a type of confirmative research conducted by collaborating centers [2]. P3PT should be performed prior to early stage clinical investigations and shall contribute to the strongly desired predictive value increase in stroke research.

Expert consortia are currently defining potential P3PT frameworks and guidelines, including ways for their future implementation. In parallel, a number of editorials, white papers, and commentaries have reviewed the concept. However, such publications exclusively represent statements by groups of selected experts or renowned individuals. While this provides well-thought through and highly relevant impulses expediting and refining the P3PT idea, it omits the chance to include ideas and feedback from a broader audience.

The first P3PT has recently been completed [3], but wider adoption and sustained utilization of the P3PT concept requires acceptance throughout the community down to its grassroots, i.e., junior investigators in smaller stroke research laboratories, as well as technicians and students conducting experiments everyday. Hence, the currently ongoing, expert consortia-based design of the P3PT framework might benefit from sensory input from a diverse audience representing the wider stroke research community without balancing towards a specific subgroup.

## Collecting a Community Feedback on the P3PT Concept

We sought community feedback on the P3PT concept by a public call for an online questionnaire which was announced in a previous publication [4]. The roster contained single (SA) and multiple answer (MA) questions plus five free text answers (see supplementary information). It was hosted by SoSci Survey (Munich, Germany) for 6 months. Answers were assessed for repeated access to avoid bias from counting multiple, but similar and potentially extreme statements. Received information was further subjected to a plausibility check to ensure information consistency (see supplementary material for details). Only those contributions addressing an a priori defined minimum of one survey section were included in the final analysis. An exception was a negative statement regarding general acceptance of the P3PT concept (first question), which was recorded even in case no further question was answered to prevent missing any potential negative statements on the concept. All survey questions, methodological details on the feedback acquisition strategy and data analysis are given in the supplementary material, which also contains complete collection of all free text answers.

Of note, the survey was designed as a completely anonymous platform and did not weight individual contributions by the responder’s level of responsibility, experience, or visibility in the field. Nevertheless, the feedback received on the free text answers suggests that a significant proportion of individuals responding to our call are experienced scientists, having profound experience with clinical research, and/or oversee a wide spectrum of research activities. A total of 93 contributions were considered for analysis based on aforementioned plausibility checks, with 81 individuals completing the entire survey.

## The Community View on P3PT Organization and Quality Assurance

**Fig. 1:**
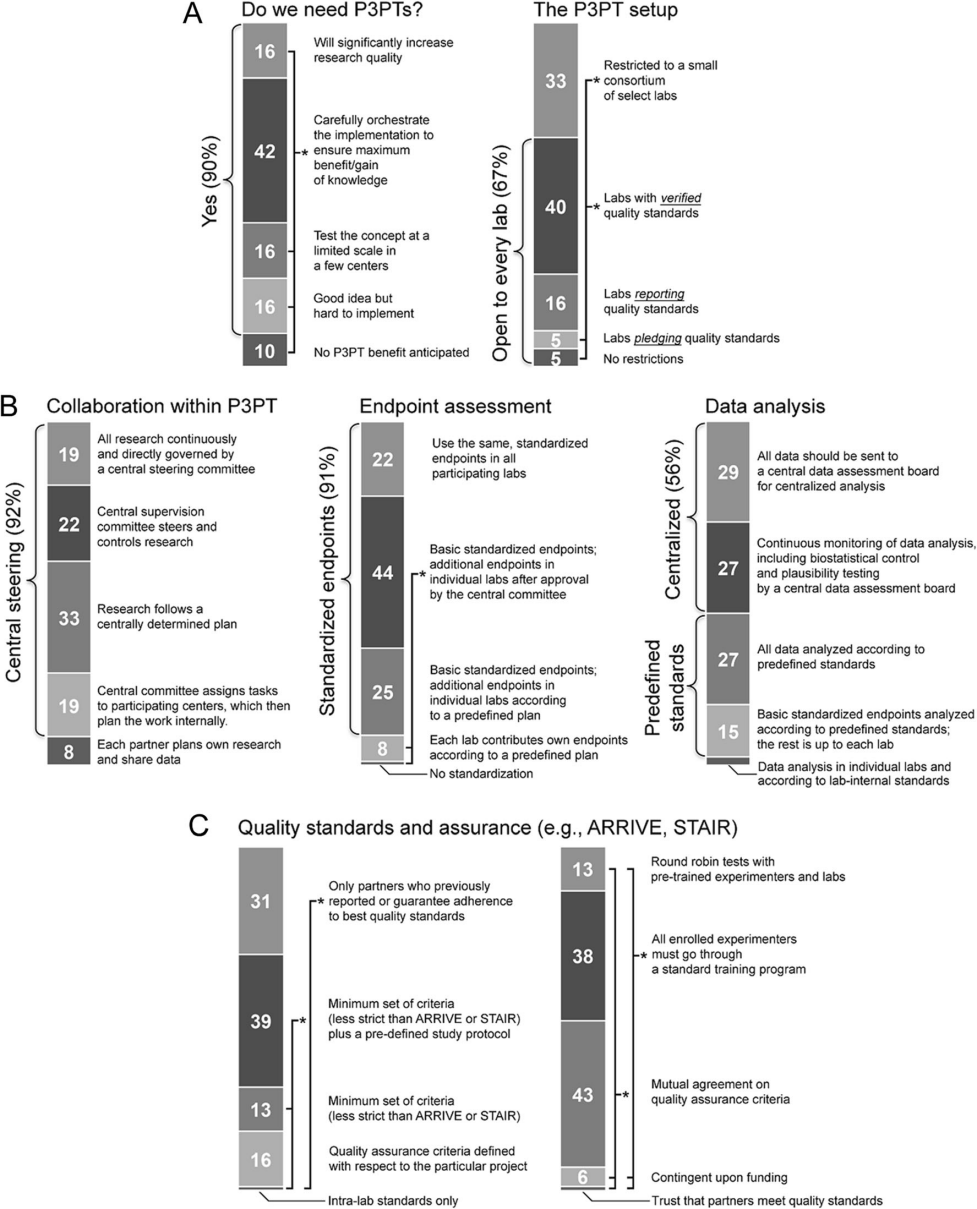
Feedback on P3PT organization and quality assurance as provided by survey participants. **a**, **b** provide answer frequency on questions regarding organization while **c** shows community statements regarding quality standards and their assurance in P3PTs. *p < 0.05

Overall acceptance of the P3PT concept was very high (Fig. 1a). Only 10 % questioned the concept while 90 % acknowledged at least a theoretical benefit. Respondents found a clear and significant overall benefit, recommending to test the concept at a limited scale initially, or assumed it hard to implement but acknowledged its theoretical value (16 % each). The majority (42 %, *p* < 0.05) requested a careful implementation to ensure maximum benefit. We support this position because careful and potentially stepwise implementation of the concept is warranted in order to investigate which organizational items provide the best balance between practicability and study design complexity, and mitigate the risk of larger failures.

The application of high quality standards seems mandatory to ensure a maximum benefit from P3PT. Accordingly, most participants recommended restricting P3PT projects to centers evidently applying quality standards (40 %, see below for implementation-specific standards), or even to pre-selected labs (33 %, Fig. 1a). This would, however, exclude smaller and less experienced laboratories from P3PT projects at least in initial stages.

A centrally determined study protocol for each lab (33 %) was not recommended statistically more often over decentralized monitoring approaches or strict centralized surveillance (Fig. 1b). Most participants preferred a common study design with pre-set endpoints for all participating centers. This indicates a high awareness for standardization within P3PT. However, a majority of participants (44 %) also advocated for a design which, next to addressing centrally determined endpoints according to a common plan, allows additional endpoints to be investigated by individual labs (*p* < 0.05; Fig. 1b). This suggestion is remarkable since it offers an option to capitalize on benefits from a centralized study organization (e.g., reliable study results on primary endpoints) without omitting the possibility to receive valuable secondary endpoint data by utilizing individual lab competencies. Although the assessment of such secondary endpoints is statistically less powerful due to the smaller number of subjects/cases investigated, overall information content and translational relevance are likely to be increased. Importantly, this also underpins the importance of academic centers as the main stakeholders in P3PTs because other well organized and equipped entities such as commercial contract research organizations often cannot offer a similar diversity of available methods and readout assays. No clear preference was given for data analysis, but centralized data analysis (29 %) or at least analysis surveillance (27 %) were selected most often (Fig. 1b).

Application of minimum quality assurance criteria plus a pre-defined experimental plan applying to each lab (39 %), or even restricting participating labs to those applying ARRIVE (http://www.nc3rs.org/ARRIVE) or STAIR [5] criteria recommendations (31 %; *p* < 0.05 each), were considered superior to intra-lab quality assurance (Fig. 1c). Official agreement on quality assurance criteria by all partners (43 %) and standardized experimenter training (38 %) were superior to all other options, including pre-study round robin trials for quality check-up (13 %; *p* < 0.05 each; Fig. 1c).

## P3PT Methodology

**Fig. 2:**
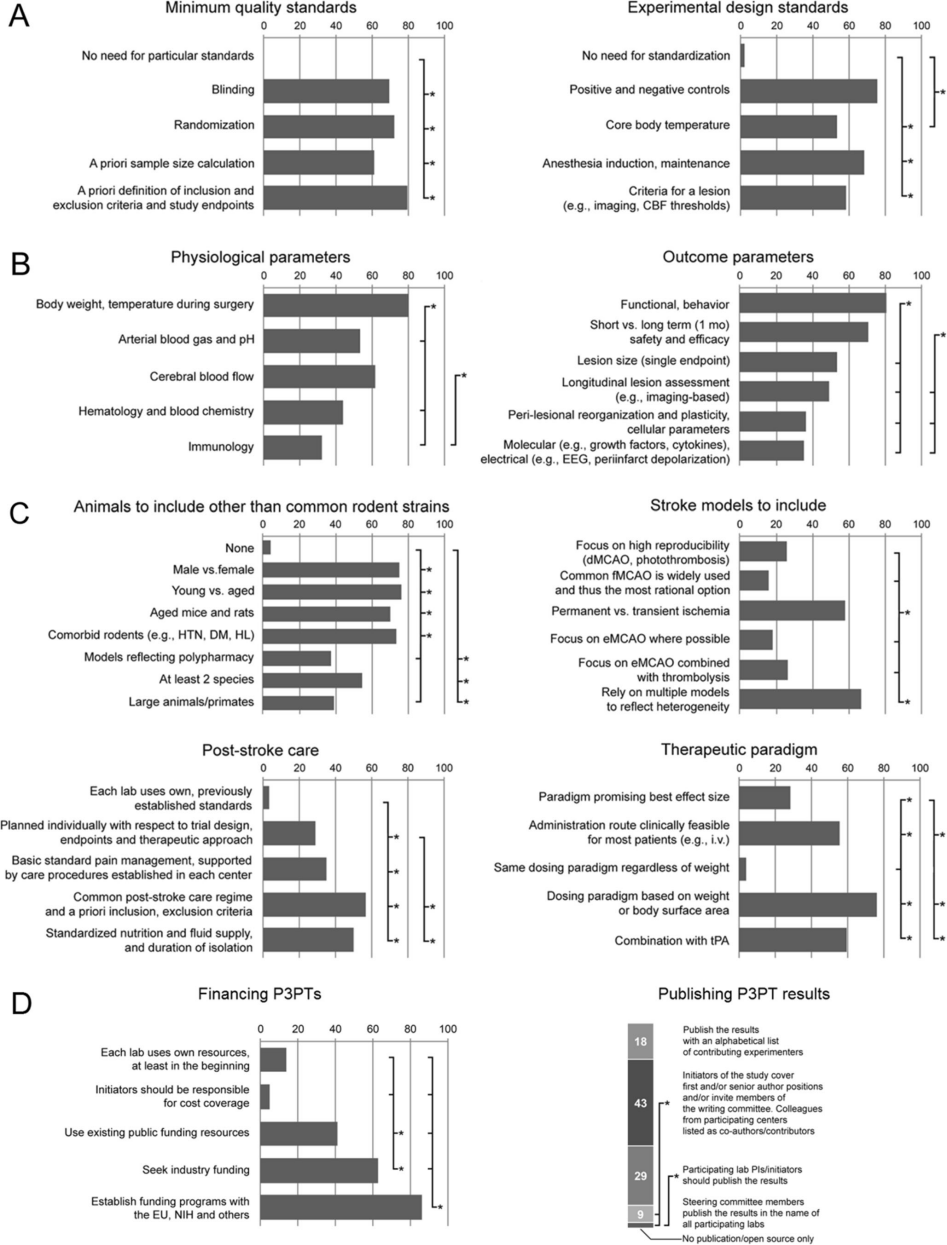
Feedback P3PT methodology, financing and result publication. **a** to **c** provide answer frequencies on questions regarding numerous central aspects of P3PT methodology. Abbreviations are as follows: *HTN* hypertension, *DM* diabetes mellitus, *HL* hyperlipidemia, *d* distal, *e* embolic, *f* filament middle cerebral artery occlusion (*MCAO*). **d** shows preferred options regarding P3PT funding and results publication. *p < 0.05

Each aspect suggested for intra-experimental quality assurance was at least recommended by 60 %, with blinding/randomization (69 %/72 %) and definition of exclusion/inclusion criteria (80 %) being selected most frequently. All were considered superior to omitting standardization (*p* < 0.05; Fig. 2a). Appropriate positive/negative controls were recommended most frequently for the experimental design (76 %; Fig. 2a), corroborating a clear community affirmation to quality assurance principles.

However, 31 % did not put special emphasis on blinding and/or randomization. This is a striking finding since (i) neglecting these aspects has been discussed to contribute to the translational failure in stroke research and (ii) blinding to avoid bias is an essential, uniformly adopted approach in clinical trials. The picture was also less consistent when it came to monitoring of important physiological parameters. Recording of body weight and temperature during surgery was found recommendable by most responders (80 %) while cerebral blood flow (CBF) monitoring to ensure stroke induction was not considered as important by 38 % of the participants. Although stroke induction can be monitored by alternative methods such as magnetic resonance imaging, those are technically more complex and less widely available. From our perspective, this indicates a need for a broader awareness of the necessity to thoroughly control stroke induction. Even fewer participants (53 %) considered arterial blood gases/pH monitoring during surgery to be necessary despite recommendations that thorough monitoring of blood chemistry parameters is critical to ensure result reliability in stroke research [5]. In summary, considering these key methodological aspects will be critical to ensure maximum predictability of P3PT studies. This likely requires increased awareness throughout the community or even mandatory.

Testing of therapeutic efficacy in aged versus young (76 %), male versus female (75 %), and in comorbid (73 %) rodent species were recommended most often to enhance the predictive value of P3PT (*p* < 0.05 each; Fig. 2c). Surprisingly, only 55 % considered investigation of at least two species, a central recommendation of the STAIR expert consortium [5]. Conducting large animal experiments (39 %) and considering polypharmacy (37 %) were recommended least often. This is understandable given the complexity of appropriate model systems and their limited availability. Nevertheless, studies utilizing large animal and polypharmacy models are important for a number of reasons. Large animal models may provide an additional benefit in the assessment of novel stroke therapies with respect to brain anatomy (gyrencephalic species) and potential distribution aspects of a pharmacological treatment (larger brain) [6] with primate models being the main expert recommendation [7]. Polypharmacy is a frequent observation in human stroke patients and an interaction between an experimental therapeutic and the patient’s medication is relatively likely [2]. Being hard to investigate by most single centers, P3PT may offer a practicable framework to address these aspects. The same applies to the heterogeneity of strokes often seen in clinical trials but rarely represented in experimental studies. Consequently, the use of multiple stroke models to reflect patient heterogeneity was found more useful than the reliance on any specific stroke model (67 %; *p* < 0.05; Fig. 2c).

Special emphasis was given on post-stroke care and preset exclusion/inclusion criteria with half of the participants also pointing at factors such as nutrition and fluid supply (Fig. 2c). Accordingly, participating colleagues also called for mimicking clinically realistic scenarios such as combination with tPA, body weight/surface-based dosing, and i.v. administration of the therapeutic agent when testing a therapeutic paradigm. Using same dose in all animals (3 %) or selecting administration protocols promising best effect size (28 %) were considered inferior approaches (*p* < 0.05; Fig. 2c).

## P3PT Financing and Publication

Financing and publication represent challenges in large scale preclinical studies. Although we expected a heterogeneous opinion spectrum, clear statements were provided by the community. The most popular option for publication was that study initiators should cover first and/or senior author positions and/or invite members of the writing committee, with all other experimenters listed as co-authors/contributors (43 %; *p* < 0.05; Fig. 2d). This supports the idea of forming writing committees as proven useful for large-scale clinical trials. Importantly, balancing transparency versus justified background interests (patents, technological knowhow) will require individual solutions, which should be informed by good practice in clinical trials.

Using (still non-existing) global funding schemes was found to be the most appropriate approach for P3PT financing (86 %; *p* < 0.05; Fig. 2d). This is consistent with the fact that stroke is a global burden requiring mobilization of global resources to counter it. Requesting industrial support was recommended by 63 % and is warranted since the pharmaceutical industry is expected to benefit, e.g., from concept falsification by P3PTs prior to significant reputational and financial losses in failed clinical trials [8]. Nevertheless, realization of global funding mechanisms may be hard to achieve while potential conflicts and intellectual property issues arising within academic-industrial partnerships demand careful consideration. Collaboration between leaders from basic research, industry, and regulatory authorities as previously proven beneficial [5] may be required to orchestrate P3PT realization.

## Information Derived from Free Text Answers

When categorizing answers regarding content and counting for answers referring to the particular aspect, three major points of interested within the participant community became evident: (i) implementation of very high quality standards in P3PT (*n* = 7), (ii) careful selection of endpoints, models, and participating labs while at the same time ensuring high interaction among P3PT participants (*n* = 10) as well as (iii) organizing P3PT close to the design of late stage clinical trials (*n* = 11) was particularly stressed.

## Limitations of the Survey

As with any voluntary participation in opinion surveys, scientists who are less supportive of the P3PT concept may not have taken the time to contribute their thoughts to our or other initiatives and thus potentially bias our results. However, it is equally possible that those who are not supportive of the P3PT idea could have taken this anonymous opportunity to express their opinions by answering the survey. We did not collect information regarding respondents’ positions (e.g., senior vs. junior) or their actual participation in preclinical stroke research and drug testing. This impedes weighting of feedbacks and positions provided with respect to experience and level of expertise of the respective respondent. On the other hand, the strictly anonymous nature may have helped to receive a broader feedback from the stroke community, which is critical for the acceptance and large-scale implementation of the P3PT concept.

## Conclusions

Despite its limitations, our survey provides profound feedback from a considerable number of individual respondents. Their feedback encourages further steps implementing P3PT studies into translational research strategies, but also highlights controversies around specific aspects of its implementation. Based on the analysis of received answers, we suggest drawing the following conclusions and recommendations with respect to five core areas (Table 1).

**Table 1:** Conclusions and recommendations for the implementation of P3PT based on community feedback analysis

Area	Conclusion/recommendation	Benefit
P3PT implementation	Careful and stepwise implementation recommended	Widespread acceptance more likely
	Initial preclinical multicenter studies should be performed by experienced centers, ideally having a long-standing history of collaboration	Swift and exact estimation of P3PT benefit under practical conditions
P3PT organization and governance	Centralized study governance and central study protocol	Clinical-trial like design, enhanced result comparability
	Core endpoints addressed by all centers according to P3PT protocol	Enhanced statistical power and higher predictability for primary endpoints
	Additional: individual endpoints addressed by single centers with outstanding competencies	Broad spectrum of translationally endpoints addressable (but no benefit for study power)
P3PT animal models	Use of multiple models, if applicable	Better representation of patient population (polypharmacy, age, sex, comorbidities)
	Use of large animal models, if available	Reflecting gyrencephalic brain structure, closer similarity to human situation
P3PT quality assurance	Further enhancing awareness for those (might require institutional support [9])	Increasing scientific rigor and result comparability, reducing divergences in relevance acknowledgment throughout the community
	Strict application of quality assurance criteria	Enhanced result comparability and relevance
P3PT financing and result publication	Establishing and recruiting of global funds	International and -continental collaboration facilitated, reduced financial burden for national public funding authorities
	Early enrolment of high-quality academic-industry collaborations	Timely involvement of key stakeholders, preventing failure of clinical trials, bolstering financial resources for P3PT
	Establishing centralized writing committees	More efficient workflow, comparability to large scale clinical trials

Since the P3PT idea can only be successful if supported by the entire stroke research community, input from other initiatives and expert committees such as the MULTIPART project (http://www.dcn.ed.ac.uk/multipart/default.htm) responding to our results are essential and helpful to shape P3PT implementation and to ensure its maximum benefit and impact.

## Electronic Supplementary Material

Below is the link to the electronic supplementary material.

ESM 1 (DOCX 34.2 kb)
