# Supplementary material for: Phase III Preclinical Trials in Translational Stroke Research: Community Response on Framework and Guidelines
Source: Transl Stroke Res. 2016 Jun 14;7:241–7. doi: 10.1007/s12975-016-0474-6 (PMC4927600; doi:10.1007/s12975-016-0474-6)
Supplement: Supplementary file 1 — (DOCX 34.2 kb) [file 12975_2016_474_MOESM1_ESM.docx]

**Supplementary material**

Boltze J, Wagner DC, Henninger N, Plesnila N, Ayata C.

Preclinical phase III trials in translational stroke research:

community response on framework and guidelines

**Control for repeated access and plausibility checks**

Individual responses were monitored for repeated access. In case repeated accesses from the same IP address were detected, only the first was counted as an individual answer. All others were neglected. This strategy was applied to prevent bias caused by individuals providing more than one contribution since such behavior may have resulted in a bias towards extremely positive or negative statements. Prevention of such bias was considered more valuable than the overall number of contributions recorded.

For plausibility checks, we controlled for statements repeatedly providing items with the same numerical order or no answer for more than five consecutive times. This was considered random selection and the particular dataset was excluded from analysis. In case of multiple choice questions, each answer was checked for conflicting and/or illogical answer structures. If that was the case, the particular answer was excluded from analysis. With the exception of the first question on overall acceptance (see main text for details), only contributions answering at least one section completely were considered for analysis.

**Statistical data analysis**

A binary, non-parametric analysis strategy was applied. Each time an answer was checked “1” was assigned, while “0” was given in case of non-selection. The Kruskal-Wallis test followed by Tukey’s test to correct for multiple comparisons was performed (SigmaPlot 11.0 software package). P-values <0.05 were considered statistically significant and indicated by asterisk. Answer frequency (%) in SA questions was presented as stacked bars while those in MA questions were depicted with single bars, rounded to the nearest whole number.

To depict statistically significant differences (p<0.05) in answer frequencies, lines with ticks referring to the respective answers were used. Ticks pointing right and being labeled with an asterisk indicate those answer(s) that was/were given significantly more often (p<0.05). Ticks pointing left and not being labeled with an asterisk indicate the answer(s) that was/were given significantly less often as compared to aforementioned ones.

**Assessment of free text answers**

Free text answers were primarily intended to offer an open forum for survey participants to address additional points of importance. Moreover, free text answers from each section were carefully screened and categorized regarding their main content/message by two independent authors (CA and JB). The frequency by which the respective category was mentioned represented the secondary endpoint and was considered pointing towards aspects of particular importance for the community. No statistical analysis was performed.

**Questionnaire on „Phase III Preclinical Trials (P3PTs)”**

Part 1: P3PT setup and organization / general statements

**1.1 Do we need P3PTs?**

1. I am not sure about the overall benefit
2. It is a valuable idea in theory, but might be hard to implement in research practice
3. Yes. We should start testing the concept at a limited scale with few centers being enrolled
4. Yes, but we should carefully orchestrate the implementation of the concept in research practice to ensure maximum benefit/gain of knowledge
5. Yes. P3PTS will significantly increase the quality of our research

**1.2 How shall we initially setup P3PT consortia?**

1. The initiative should be open to every lab which wishes to participate without restriction
2. The initiative should be open to every lab pledging minimum quality standards
3. The initiative should be open to every lab reporting minimum quality standards
4. The initiative should be open to every lab after verification of minimum quality standards
5. We should start with a small consortium of selected partners

**1.3 How should quality assurance in P3PTs be organized?**

1. Each partner lab should be allowed to use own standards
2. The quality assurance criteria should be defined with respect to the particular project
3. We need to have a set of minimum quality assurance criteria (but less strict than ARRIVE or STAIR criteria)
4. Minimum quality assurance criteria plus a pre-defined study protocol shall apply
5. Only partners who previously reported or guarantee adherence to best quality standards (e.g. ARRIVE, STAIR) should be included

**1.4 How should collaboration within a P3PT be organized?**

1. Each partner should organize their own research and share data with others
2. There should be central planning assigning tasks to participating centers. Centers organize work in own response
3. Research should follow a centrally determined plan
4. A central supervision committee should steer and control research activities
5. All research activities should be continuously and directly governed by a central steering committee

**1.5 What else do you think is important?**

Free text answer

Part 2: P3PT quality assurance

**2.1 What should be the minimum standards in P3PTs? (Multiple answers possible)**

1. We do not need particular standards
2. Blinding of investigators and model induction
3. Randomization of animals into groups
4. A priori sample size calculation
5. A priori definition of inclusion/exclusion criteria, and study endpoints

**2.2 How shall we ensure application of quality assurance criteria?**

(A) It is enough to trust all research partners

(B) Application of such criteria causes costs and should therefore depend on funding

(C) All partners shall officially agree on a quality assurance criteria

(D) All enrolled experimenters must go through a standard training program

(E) We need to perform round robin tests with pre-trained experimenters and labs

**2.3 What else do we need to consider?**

Free text answer

Part 3: P3PT methodology

**3.1 What kind of animals should we include in P3PTs apart from common rodent strains? (Multiple answers possible)**

(A) None

(B) Aged mice and rats

(C) Co-morbid rodents (e.g. hypertension, diabetes, hyperlipidemia)

(D) Animal models reflecting polypharmacology

(E) Large animal / primate models

**3.2 What kind of stroke models should we use? (Multiple answers possible)**

(A) We should focus on high reproducibility (distal MCAO, photothrombosis)

(B) Common filament models are widely used and thus the most rationale option

(C) We should focus on embolic stroke models where possible

(D) We should focus on embolic stroke models being combined with thrombolysis

(E) We should rely on multiple models to partly reflect heterogeneity

**3.3 Which physiological parameters should be assessed? (Multiple answers possible)**

(A) Weight and temperature before/during surgery

(B) Blood gases and pH

(C) Cerebral blood flow during model induction

(D) Hematological parameters and detailed blood chemistry

(E) Immunological parameters should be monitored

**3.4 Which of the following aspects should be standardized? (Multiple answers possible)**

(A) We do not need standardization

(B) Use of positive and/or negative controls for a particular stroke model used

(C) Core body temperature

(D) Methods to induce, maintain, and control anesthesia

(E) We need to have standardized criteria for a lesion (e.g. based on imaging or CBF thresholds)

**3.5 Which standards shall apply to post-stroke care procedures?**

(A) Each lab shall use own and previously established standards

(B) This should be planned individually with respect to trial design, endpoints and therapeutic approach

(C) We shall apply a standardized basic pain management regime supported by individual care procedures established at individual centers

(D) We shall apply a common post-stroke care regime and preset inclusion and exclusion criteria

(E) As D, but we probably will also have to consider aspects such as nutrition, fluid supply, duration of any isolation period

**3.6 When using a therapeutic agent, how should we consider its application and dose? (Multiple answers possible)**

(A) We should use administration promising best effect size

(B) We should focus on administration routes clinically feasible for most patients (e.g. i.v.)

(C) We should apply same doses to all animals regardless of body weight

(D) We should apply a body-weight and/or -surface adjusted dose

(E) We should consider combination therapies with tPA

**3.7 What should be preferred outcome parameters? (Multiple answers possible)**

(A) Functional status / behavior phenotyping

(B) Lesion size (single endpoint)

(C) Longitudinal assessment of lesion development (e.g. imaging-based)

(D) Peri-lesional reorganization and plasticity, cellular parameters

(E) Changes in molecular (e.g. growth factors, cytokines) and/or electrophysiological parameters (e.g. EEG, perilesional depolarization)

**3.8 How shall we conduct endpoint parameter assessment?**

(A) We do not need to standardize assessment; each lab should contribute to its best ability

(B) Each participating lab shall contribute with its individual expertise according to an a priori defined plan. No need for endpoints homogenously addressed in all labs

(C) We need common and standardized (basic) assessments. In addition, each participating lab shall contribute with its special competences according to an a priori defined plan

(D) We need common and standardized (basic) assessments. Additional endpoints may be addressed by individual labs with special expertise after approval by the central P3PT supervision committee

(E) We should use the same, standardized endpoint parameter assessments in all participating labs

**3.9 How shall we conduct data analysis?**

(A) Each lab shall analyze its data according to own standards and experience

(B) A number of basic tests conducted in all labs shall be analyzed according to a priori agreed standards; the rest is up to the participating lab

(C) All data shall be analyzed to pre-set standards

(D) A central data assessment board shall continuously monitor data analysis, including biostatistical control and plausibility testing

(E) All data should be sent to a central data assessment board for centralized analysis

**3.10 What other aspects are important to cover? (Multiple answers possible)**

(A) Male versus female animals

(B) Young versus aged animals

(C) At least using 2 different species enrolled

(D) Permanent versus transient vessel occlusion

(E) Short term versus long-term (at least 1 month surveillance) safety and efficacy

**3.11 What other points and aspects are of utmost importance and need to be considered?**

Free text answer

Part 4: Financing and publication

**4.1 How shall P3PTs be financed? (Multiple answers possible)**

(A) Participating labs should use own resources, at least in the beginning

(B) Initiators should be responsible for cost coverage

(C) We should use existing public funding resources to cover P3PTs

(D) We should try to enroll industry funding

(E) We should try to establish funding schemes for P3PTs together with the EU, NIH, and other authorities

**4.2 How should we publish results from P3PTs?**

1. We should not publish results/only publish in media open to a large community (homepages etc.) in order to avoid bias
2. The steering committee members should publish the results in the name of all participating labs
3. The participating labs’ PIs/initiators should publish the results
4. Initiators of the study should cover first and/or senior author positions and/or invite members of the writing committee. Colleagues from participating centers shall be listed as co-authors/contributors
5. We should publish the results with an alphabetic list of contributing experimenters

**4.3 What else do you think is important?**

Free text answer

Part 5: General comments, critique and recommendations

Please provide any comments or recommendations you find important that have not been covered by this survey.

**Collection of all provided free text answers**

All answers are reproduced as provided and were not edited for wording or content. Obvious typos have been corrected. No answer was excluded, even if the relation to the question and survey part was not obviously clear.

Free text answers to Part 1: P3PT setup and organization / general statements

Question: What else do you think is important?

*“A balance; the activities should be controlled however, the participating researchers should have the possibility to be involved as part of a team”*

*“Solicit ideas from participating labs and agree on 3-4 ideas. Then each lab should test these ideas on a specified study protocol”*

*“mimic clinical trials as much as possible: central data storage and analysis, pre-defined study plan, etc.”*

*“This is an outstanding initiative, and much needed to move high quality research forward. However, I do not believe that such rigorous standards should apply to high-impact, preliminary studies that are observational in nature.”*

*“Maybe it is not necessary to include a large number of laboratories, but better a small number of dedicated laboratories. There are European reference laboratories for other areas, so maybe EU can also provide funding in the P3PT project (because it will need considerable resources for the laboratories involved)”*

*“There are many aspects to P3PT that are important including quality standards, assessment criteria, funding, data management, and choice of participating centres. All of these will have to be rigorously thought through, and dealt with before P3PT goes ahead.”*

*“As currently proposed, the idea of a Phase III pre-clinical research platform is expensive. It carries a considerable administrative overhead, for example. Research funding is tight in most countries. There is really no way of funding a top-down, administratively”*

*“Frequent feedback and discussions on progress of experiments, practical issues, etc. between involved partners”*

*“We should organize based on the typical organization of large scale multi-center clinical trials. Rather than 'reinventing the wheel', we can use their lessons and successes to guide our organization.”*

*“Quality standards should definitely go beyond ARRIVE or STAR.”*

*“Regular meeting and hands-on training should be organized.”*

*“Selection of the involved research groups should be fair. Funding to be provided to the selected groups to perform the studies (how? by who?)”*

*“The study plan should take benefit of the models available in individual labs.”*

*“Limit the number of stroke models and have thought about which ones to choose depending on the study design.”*

*“steering committee would probably need to be independent from the study as in a clinical trial”*

*“Randomization or dispersion of the diversity variables so that there is not an interaction between variable and center. A weak center could create a false positive or negative finding. High quality at all centers is important. In addition to initial quality check for entry, suggest considering an on-going quality program. To use analogy to clinical trials, site monitor visits or audits should be conducted periodically.”*

*“The roadmap here should be pre-determined including protocols, stat-plan etc as one would do for a typical clinical trial”*

*“regular meeting with official representatives from every collaboration partner”*

*“Research shows that well designed Phase II trials paved path for various successful phase III trials and approved drugs in cancer research.*

*Why Do So Many Phase 3 Clinical Trials Fail?Part 1: The Effect of Deficient Phase 2 Trials in Therapeutic Areas with High Failure Rates in Phase 3 Studies*

*By: Anastassios D. Retzios, Ph.D.* [*http://adrclinresearch.com/Issues_in_Clinical_Research_links/Why%20Pivotal%20Clinical%20Trials%20Fail%20-%20Part%201_v12L_a.pdf*](http://adrclinresearch.com/Issues_in_Clinical_Research_links/Why%20Pivotal%20Clinical%20Trials%20Fail%20-%20Part%201_v12L_a.pdf)*”*

*“Community buy-in (including patients and providers). Nursing buy-in (they are the largest work force and will likely be implementing the clinical trials as coordinators.”*

Free text answers to Part 2: P3PT quality assurance

Question: What else do we need to consider?

*“Since the number of animals that will be involved in the P3PT will be large, there is no need for high reproducibility of the experimental model. Therefore, original models, more closely mimicking the human pathophysiology should be performed. For example: direct embolization of in situ formed thrombi (induced by FeCl3 for instance) from the common carotid artery, which lead to large/small profound/superficial cortical/striatal/hippocampal lesion is particularly interesting. The intrinsic variability of this kind of model precludes its use in single center study (sample size calculation leads to a "n" of ~450 according to our own results) but in large P3PTs this would be feasible and particularly relevant.”*

*“alternative stroke models with disease and aged rats”*

*“If animals are excluded due to premature death or model failure (particularly small stroke) this data should be reported.”*

*“Scheduled auditing of data coming out of each lab and if there is significant deviation from one lab then explanation should be sought and their protocols should be evaluated.”*

*“Maybe even collaboration with companies which ensure quality in clinical trials”*

*“Assessing quality of centre by looking at previous publications and how well they follow quality standards. I do not think site visits should be necessary as these will be prohibitively costly and invasive to the lab. Site visits should only be conducted when something is amiss with the data produced by one lab.”*

*“training should be offered, but does not necessarily need to be mandatory (assuming the participating centers know what they are doing in the first place)”*

*“The definition of quality criteria will depend on the complexity of the procedures required for disease induction, treatment and endpoint evaluation.*

*Preferably simple procedures with robust outcome parameters should be used in the initial P3PT trials.*

*Funding will be an important issue to assure success.”*

*“Maximal genetic heterogeneity of subjects (e.g. as in the NIA ITP)”*

*“Site visits as in clinical trials”*

*“Not convinced that any of these can ensure QA”*

*“Ensure quality assurance with the same methods used in phase III clinical trials. Same requirements for data monitoring and personnel training. “*

*“Replication of data for the various co-morbidities, not just the therapeutic window”*

*“operate in accordance to harmonized SOP´s”*

*“Negative data should be reported; publication bias should be avoided.”*

*“Importance of aseptic technique during surgery”*

Free text answers to Part 3: P3PT setup and organization / general statements

Question: What other points and aspects are of utmost importance and need to be considered?

*“Negative results should be published.”*

*“For some models (e.g., embolic) long term survival may not be feasible.”*

*“comorbidities”*

*“I don’t do animal research at this point but would be interested if an opportunity is there”*

*“It would be good to have a pool of standardized models. A committee would decide which models and endpoint parameters are appropriate for the drug and the indication (e.g. stroke: certainly aged animals important; for traumatic head injury may also very young animals necessary)”*

*“The problem with P3PT will be that there are so many variables to consider that no one trial will be able to tackle them all. These variables such as model, dose, species, anesthesia, age, sex, physiological monitoring are all very important and have all been shown to affect outcome, but I think each trial's parameters will depend on the therapeutic agent being tested.”*

*“I think we will have to go through a two-step process: First using very standardized models to demonstrate reproducibility across labs and the feasibility of the design.*

*the highest clinical impact will probably be achieved if a broad variety of models responds to the same intervention.”*

*“- Discussion and agreement on appropriate treatment dosage, frequency, administration, etc.*

*- Co-morbidities (hypertension, hyperglycemia, etc.)”*

*“Endpoints, physiological monitoring and other standards will differ from acute neuroprotection studies to neurorepair and recovery projects; different guidelines need to be established.”*

*“It is useful to have individual labs analyze their data to pre-set standards, but also have a centralized data assessment board. A lot can be learned from this.”*

*“Not convinced that aged rodents help us much.”*

*“Common comorbidities such as hypertension”*

*“Trial design should follow as closely as possible the design and analysis methods for typical clinical stroke trials, or better yet, the anticipated design/methods of the clinical trial envisioned for the therapy under question.”*

*“The testing of comorbidities, age and sex should be done with the best therapeutic window determined. The therapeutic window may not be the same for someone with hypertension versus someone with hypertension, diabetes and hypercholesterolemia”*

*“Drug metabolism often ignored between animal vs. humans; drug levels need to be measured in the brain to demonstrate the drugs are reaching the brain (not with an assumption that since effects are seen drugs are reaching the target brain tissue).”*

*“co-morbidities such as hypertension and hyperglycaemia and type II diabetes”*

Free text answers to Part 4: Financing and publication

Question: What else do you think is important?

*“Congratulations on this interesting and probably trend-setting approach in preclinical stroke research!”*

*“Novel relationships with industry and healthcare institutions”*

*“The funding question deserves clarification. Participating labs should be responsible for having the infrastructure to conduct trials including the ability for physiological monitoring. However, using aged or comorbid animals may be cost prohibitive and be covered through the sponsor. Central analysis of data should be covered.”*

*“What about potential intellectual property resulting from such studies?”*

*“If P3PT would be within the frame of a European Reference Center, a steering committee of the participating centers may also be the initiators.”*

*“Financing these trials will be difficult. Until the first trial is conducted that shows applicable translation to the clinic, then not many funding agencies including industry will support this. I believe, once industry are on board, funding will become easier but this won't happen until the results from P3PTs have been shown to be translatable.”*

*“This section brings up the biggest issue in this approach: cost. Industry will not participate because of the lack of control and I.P. issues. Individual labs cannot take these projects on their own funding resources, which are stretched thin. If existing funding sources are re-apportioned to this kind of platform, it will diminish stroke funding from these sources for other projects. The funding of a large administrative bureaucracy for planning, standardization, data analysis, etc. will meet with widespread criticism in the general science community.”*

*“This should be a global effort including Asia.”*

*“Authorship depends also on the funding: if the lab PI needs to find the funding himself, she should be a coauthor. If the study is paid for, he should be listed as a collaborator.”*

*“PhD students and post-docs may spend a short period of time (e.g., 4 weeks) in the other labs involved in the project to compare the procedures and to establish feasible common standards.”*

*“Oxidative stress after experimental stroke is considered as one of significant parameter and several agents targeted this issue and failed (NXY-059 etc.). In the clinic the clotbuster tPA after dissolving the clot restores blood flow, which can initiate oxidative stress; are the stroke patients after treatment with tPA are followed up any antioxidant treatment?“*

*“Open access publications”*

Part 5: Any comments (free text answer)

*“The experimentation should be performed by skilled lab staff members and not students.”*

*“I think it is a great idea and there is a need for collaboration at the basic sciences level on the model of clinical trials.”*

*“If there will be an assignment from industry to test certain compounds the intellectual property and the profit certainly remains with the company.”*

*“P3PTs are a great idea, but they are full of logistical hurdles and will be difficult trials to conduct, particularly in the beginning. They should not replace the single centre safety, efficacy and mechanistic studies that are being carried out. I hope that P3PTs go ahead and they are shown to be predictive of clinical trial results but I suspect it will be many years before this will occur. I commend the Stroke Research community for driving this idea forward, and the Multi-PART consortium appears well on their way to setting up a P3PT for stroke.”*

*“Differences between animal welfare protocols/requirements between different continents need to be carefully assessed. Animal variations between different continents have to be factored-in.*

*The outlook and needs of the labs in different continents are sometimes very different. For example, investigators in USA are under pressure to conduct studies and publish faster than their European counterparts for financial reasons.”*

*“We should consider including in these studies laboratories that are not yet at ARRIVE guidelines and may have the opportunity to improve their standards.”*

*“Studies on neuroprotection and recovery enhancing therapies are equally important.“*

*“Animal protocol approval. In Switzerland, to perform an experiment, you must show that it is novel. At present, you can't get approval to repeat an experiment...”*

*“The organization and implementation will require careful thought to get it off the ground”*

*“If it is not possible to power the study for subgroup comparisons (e.g. young vs. old) suggest using the characteristics of the human stroke population as a template for the rodent population. This would mean mostly aged animals, significant proportion with hypertension, significant portion with diabetes, etc.”*

*“Many public private partnerships already exist for neurodegenerative diseases such as CAMD, CCNA and others. Learn from them before embarking in such an exercise.”*

*“Importance of engaging with the public so that they are aware of this type of initiative and can support it through social media etc.”*
